# Identification and Validation of Promising Targets and Inhibitors of Biofilm Formation in *Pseudomonas aeruginosa*: Bioinformatics, Virtual Screening, and Biological Evaluation

**DOI:** 10.3390/pathogens14090855

**Published:** 2025-08-28

**Authors:** Ting-Ting Liang, Ju-Qi Wen, Ge-Ping Chen, Rui Wang, Jun Xu, Wen-Ying Chen

**Affiliations:** 1 Department of Pharmacy, The Third Affiliated Hospital, Southern Medical University, Guangzhou 510630, China; tingtingliang2022@163.com (T.-T.L.); chengeping2024st@163.com (G.-P.C.); 18211256090@163.com (R.W.); 2 College of Pharmacy, Jinan University, Guangzhou 510632, China; wenjuqi97@stu.jnu.edu.cn

**Keywords:** GacS, bioinformatics analysis, docking, molecular dynamics simulations, biofilm, *Pseudomonas aeruginosa*

## Abstract

*Pseudomonas aeruginosa*, a member of the “ESKAPE” group of bacterial pathogens, exhibits biofilm-forming capacity, a key factor contributing to its resistance to conventional antibiotics and posing significant challenges in clinical treatment. To develop more effective therapeutics against such infections, identifying potential drug targets through bioinformatics analysis is essential. Consequently, we utilized data from the GEO database to investigate differentially expressed genes between planktonic and biofilm groups, and identified drug targets through the construction of a protein–protein interaction (PPI) network and the cytoHubba algorithm. Inhibitors targeting this protein were identified through molecular docking screening of the FDA-approved drug library, and their anti-biofilm activity was validated in vitro. Through bioinformatics analysis, we identified GacS as the drug target in this study for treating biofilm-related infections. Virtual screening revealed that oxidized glutathione (GSSG) and arformoterol tartrate (ARF) are both capable of tightly binding to GacS and demonstrating good stability. In vitro experiments further confirmed that both GSSG and ARF demonstrated anti-biofilm activity, particularly when combined with azithromycin (AZM) or clarithromycin (CAM), significantly enhancing the biofilm inhibition effects of these antibiotics. This combination therapy offers a new and innovative strategy to combat biofilm-associated infections, showcasing the potential of GacS inhibitors in clinical applications. In conclusion, GSSG and ARF may serve as effective GacS inhibitors, and their combination with AZM or CAM could provide a novel approach for treating biofilm-related infections, paving the way for more effective treatment options.

## 1. Introduction

*Pseudomonas aeruginosa* is a notorious opportunistic pathogen responsible for severe acute and chronic infections, particularly in immunocompromised individuals, such as those with cystic fibrosis or burn injuries [1,2,3]. The high persistence of this species in clinical settings is primarily due to its capacity to develop antibiotic-resistant biofilms [4]. Bacteria within biofilms can be 10 to 1000 times more resistant to antibiotics than their planktonic counterparts, primarily due to the inability of antibiotics to penetrate the complex polysaccharide matrix. Additionally, biofilms exhibit altered metabolic activity and protein synthesis, further contributing to antimicrobial resistance [5,6,7]. Up to 70% of chronic wound infections involve bacterial biofilm barriers, rendering antibiotics ineffective [8]. Within the United States, approximately 6.5 million patients are affected by chronic wound infections over the long term, leading to over USD 25 billion in annual healthcare costs [9]. These challenges highlight the urgent need to develop therapeutic strategies that target biofilm formation.

Among the key regulatory mechanisms involved in biofilm development, two-component systems (TCSs) play a central role [10]. A typical TCS consists of a histidine kinase and a response regulator that together sense environmental signals and modulate diverse bacterial functions [11]. Because TCSs are widely conserved in bacteria but absent in mammalian hosts, they are considered promising targets for antimicrobial therapy. In *P. aeruginosa*, several TCSs, such as GacS/GacA, FleS/FleR, and ChpA-PilG, have been shown to regulate biofilm formation, virulence, and motility [12].

In recent years, bioinformatics analysis has become an essential tool for identifying key genes and pathways associated with disease mechanisms, allowing researchers to efficiently screen novel biomarkers and therapeutic targets [13,14]. By leveraging bioinformatics analysis of gene chip data from *P. aeruginosa* biofilm samples, we identified GacS as a promising therapeutic target for combating biofilm-associated infections. GacS, a histidine kinase formerly known as LemA, together with the response regulator GacA, forms a non-canonical two-component system (TCS) that regulates quorum sensing, biofilm maturation, and chronic infection via the Gac/Rsm signaling cascade [15,16]. Although several studies have highlighted the biological role of GacS/GacA, research on its inhibition remains scarce. In 2013, Dieter Haas discovered that azithromycin blocked the expression of the *rsm* gene in this system, primarily by inhibiting key activators during their transcription process [17]. Additionally, Carlson et al. demonstrated that the 2-aminobenzothiazole scaffold inhibits the phosphorylation of GacS/GacA, showing histidine kinase inhibition and antibacterial activity. These findings support the potential of GacS/GacA as a therapeutic target. However, there are currently few reports on GacS inhibitors, and a lack of virtual screening models targeting this system [18].

In parallel, combination therapy involving non-traditional and conventional antibiotics has gained increasing attention. Such approaches not only reduce antibiotic usage but also enhance the inhibition of biofilm formation, leading to improved treatment outcomes.

Recent studies have focused on the development of anti-biofilm agents that target quorum sensing (QS) systems and iron homeostasis, two key regulatory pathways involved in *P. aeruginosa* pathogenicity [19,20,21,22]. In order to identify more promising targets related to biofilm formation, we used bioinformatics approaches to identify differentially expressed genes (DEGs) related to *P. aeruginosa* biofilm formation in real bacterial samples; the genes were identified via a search of the Gene Expression Omnibus (GEO) database. Subsequently, we constructed protein–protein interaction (PPI) networks to identify key hub genes as the target. Furthermore, in an effort to repurpose existing drugs, we performed virtual screening of the FDA-approved drug database to identify potential inhibitors and explored optimal combination therapy strategies with azithromycin (AZM) and clarithromycin (CAM).

## 2. Materials and Methods

### 2.1. Data Sources

The GEO database (National Center for Biotechnology Information, https://www.ncbi.nlm.nih.gov/geo/, accessed on 15 July 2023) was searched using the keywords “*Pseudomonas aeruginosa*” AND “biofilm” to identify suitable datasets for analysis. Initially retrieved datasets were screened based on the following explicit inclusion criteria: (1) datasets must contain both biofilm-forming and planktonic growth conditions for direct comparison; (2) each group must include at least two biological replicates to ensure statistical robustness; and (3) experiments must be conducted under untreated conditions without antibiotics, chemical treatments, or genetic modifications to avoid confounding effects. Datasets not meeting these criteria were excluded. Ultimately, the dataset GSE10030 (Affymetrix al. found that biofilm formation was significantly reduced in *Pseudomonas aeruginosa* array, GPL84), published by Anderson et al. [23], was selected. This dataset included samples from *P. aeruginosa* grown as biofilms (samples GSM252496, GSM252501, GSM252505) and in planktonic state (samples GSM252559, GSM252560). Antibiotic-treated samples within the dataset (GSM252506, GSM252507, GSM252508, GSM252561, GSM252562) were excluded. As this dataset was publicly available, no local ethics committee approval was required.

### 2.2. Data Preprocessing

Information related to DEGs associated with biofilm formation (BF-DEGs), including gene ID, gene name, *p*-value, adjusted *p*-value, and logFC, was obtained from the dataset by using the “limma” package in R software (version 4.3.0). Genes with incomplete information were excluded, and those with a *p*-value < 0.05 and |logFC| > 1 were selected for detailed analysis of differential expression.

### 2.3. Gene Ontology and Pathway Enrichment Analysis

To further understand the biological role and significance of differential gene expression, we utilized the DAVID database (https://davidbioinformatics.nih.gov/) to conduct Gene Ontology (GO) and Kyoto Encyclopedia of Genes and Genomes (KEGG) pathway enrichment analysis [24,25,26]. The analysis results with *p*-value < 0.05 and count > 8 were visualized.

### 2.4. Construction of a Protein–Protein Interaction (PPI) Network and Identification of a Hub BF-DEGs

In order to investigate protein interaction and function, differential genes were uploaded to the STRING database (https://string-db.org/, accessed on 15 July 2023), based on a minimum interaction score of 0.4 and excluding disconnected nodes from the network. Subsequently, Cytoscape software (version 3.10.1) was applied to visually represent the protein interaction network (PPI). Within the PPI network, different proteins exhibit varying degrees of connectedness. The central nodes with high connectivity may represent core or key proteins, which have the potential to become biomarkers or drug targets. Using the cytoHubba plugin [27], the top 10 genes were identified by applying algorithms such as Betweenness, MNC, Closeness, Degree, and EPC; the resulting overlap indicated hub genes related to biofilm formation.

### 2.5. Analysis of Functional Roles and Signaling Pathways of Hub Genes

Based on the KEGG PATHWAY database, we analyzed the top 10 hub genes identified by the Degree algorithm, focusing on their roles in metabolic pathways, signaling pathways, and biological processes. Through visualization of protein–protein interactions and associated gene expression processes, we elucidated the intricate signaling networks governed by these hub genes that regulate dynamic molecular changes in *P. aeruginosa* biofilms.

### 2.6. Preparation of Proteins and Ligands

The three-dimensional structure of GacS (PDB ID: 5O7J) was retrieved from the RCSB Protein Data Bank (https://www.rcsb.org/, accessed on 28 December 2023). The SAVES platform (http://services.mbi.ucla.edu/SAVES/, accessed on 28 December 2023) was used to generate a Ramachandran plot to assess the structural quality of the protein. Subsequently, we performed some necessary structural preparations of the target protein. These included the addition of hydrogen, deletion of water molecules, and energy minimization, which were conducted using the OPLS4 force field in the Schrödinger protein preparation wizard [28]. These options were set to their default values. A total of 1729 ligand molecules was sourced from the FDA-approved drugs database (Topscience, Shanghai, China). The ligands were prepared using the LigPrep module with the appropriate parameters, including energy minimization and the generation of tautomeric states, which were carried out using the Epik method at pH 7.0 ± 2.0 [29].

### 2.7. Generation of the Receptor Grid

The periplasmic detector (GacSPD) domain of GacS plays a critical role in the function of GacS. A *gacS* mutant lacking the periplasmic detector domain shows a significant deficiency in biofilm formation. Ali-Ahmad A et al. have used NMR spectroscopy to characterize the protein structure of GacS, revealing a unique PDC/PAS α/β fold, consisting of a three-stranded β-sheet flanked by α-helices and accompanied by an unusual major loop [30]. Arg94, His97, His124, and solvent-exposed His133 are key residues in a putative ligand-binding site; mutations of these residues lead to severe defects in biofilm formation and altered RsmY/RsmZ expression [30]. Accordingly, we define the active cavity formed by the four important residues Arg94, His97, His124, and His133 as the docking pocket.

### 2.8. Molecular Docking

Molecular docking was performed based on ligand–protein interaction using the Glide module [31]. Through two-stage molecular docking comprising Standard Precision (SP) primary screening and Extra Precision (XP) refinement, we identified the top 10 scoring compounds. Subsequent structural cluster analysis selected representative compounds from each cluster for further investigation.

### 2.9. Molecular Dynamics Simulation Analysis

Several modules of the Desmond software (version 2023-1) were used for molecular dynamics simulations, including the System Builder, Minimization, Molecular Dynamics, and the MMGBSA module in Prime [32]. First, the System Builder was used to create a water environment, where the complex was dissolved, and Na^+^ and Cl^−^ ions were added to balance the charges. The Minimization module was then used to perform 500 ps of energy minimization on the docking energy of the entire system to ensure its stability.

The formal molecular dynamics simulation was carried out using the Molecular Dynamics module, with the simulation time set to 100 ns and approximately 1000 frames. Additionally, using the thermal_mmgbsa.py script provided in the Schrödinger system, we evaluated the average binding free energy of the complex under steady-state conditions.

### 2.10. Bacterial Strains, Media, and Culture Conditions

The strain of *P. aeruginosa* used in this study was PAO1, which was stored at −80 °C in a ceramic bead culture storage tube. Before each experiment, the strain was inoculated onto an LB agar plate and incubated overnight to obtain single colonies. A single colony was selected and incubated overnight in LB broth at 37 °C.

### 2.11. Determination of Minimum Inhibitory Concentration (MIC)

The minimum inhibitory concentration (MIC) of the candidate drug was determined through the microdilution method. On a sterile ultra-clean bench, 100 μL of MH broth and 100 μL of candidate compounds were added to each well of a 96-well plate, followed by a two-fold dilution. The bacterial suspension was then added to the 96-well plate using a pipette, and the plate was incubated at 37 °C for 16 to 20 h. All experiments were conducted a minimum of three times. The MIC was defined as the lowest drug concentration that completely inhibited visible bacterial growth after incubation.

### 2.12. Biofilm Quantification by Crystal Violet (CV) Assay

Overnight cultured *P. aeruginosa* was diluted with ABTGC medium (composed of 0.1% MgCl_2_, 0.1% CaCl_2_, 0.1% FeCl_3_, 10% A10 buffer, 0.2% glucose, and 0.2% casamino acids) to an OD600 of 0.02 and then added to 96-well plates. The operation was performed on a sterile super-clean bench with 100 μL of drug and 100 μL of diluted bacteria for the experimental group. The Blank group (medium only) and the Control group (bacteria and medium) were set up at the same time. The 96-well plate was incubated in a static culture chamber at 37 °C for 24 h. After the bacterial suspension was removed, it was washed three times with PBS, after which 200 μL of methanol was added to each well, and the mixture was fixed at room temperature. Following this, the biofilms were stained with 0.1% crystal violet for 15 min and then rinsed with distilled water. The stained dye was dissolved in 30% acetic acid, thoroughly mixed using a microplate shaker, and the absorbance was measured at a wavelength of 570 nm using a microplate reader. The absorbance of the blank group was subtracted from the OD values of the control and experimental groups to correct for background and non-specific staining. Experiments were repeated at least three times.

The inhibition rate of biofilm formation was calculated using the following formula:

$$Inhibition\;rate\left(\%\right)=\frac{\left({OD}_{Control\;group}-{OD}_{Blank\;group}\right)-\left({OD}_{Experimental\;group}-{OD}_{Blank\;group}\right)}{\left({OD}_{Control\;group}-{OD}_{Blank\;group}\right)}\times 100\%$$

All statistical analyses were performed using GraphPad Prism 9. Differences between the combination and antibiotic-only groups were evaluated using two-way ANOVA, followed by Fisher’s least significant difference test for multiple comparisons. The significance threshold was set at *p* < 0.05.

## 3. Results

### 3.1. Identification of DEGs Related to the Formation of Biofilms (BF-DEGs)

**Figure 1:**
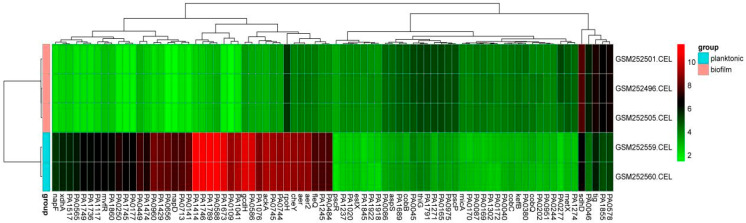
Heatmap displaying the top 80 differentially expressed genes (DEGs) based on adjusted *p*-value and logFC. Higher gene expression is shown in red; lower gene expression is shown in green.

In this study, we retrieved the biofilm-related microarray expression profiling dataset GSE10030 for *P. aeruginosa* from the GEO database. Comparative analysis between planktonic and biofilm groups identified 1098 significantly differentially expressed genes (DEGs). A smaller adjusted *p*-value indicates a higher level of statistical significance and a higher ranking of the gene among the identified DEGs. The top 40 up- and down-regulated DEGs from the incorporated data are displayed individually in Table 1. A heatmap showing 40 up-regulated and 40 down-regulated DEGs is presented in Figure 1.

### 3.2. GO Term and KEGG Pathway Enrichment Analysis of BF-DEGs

**Figure 2:**
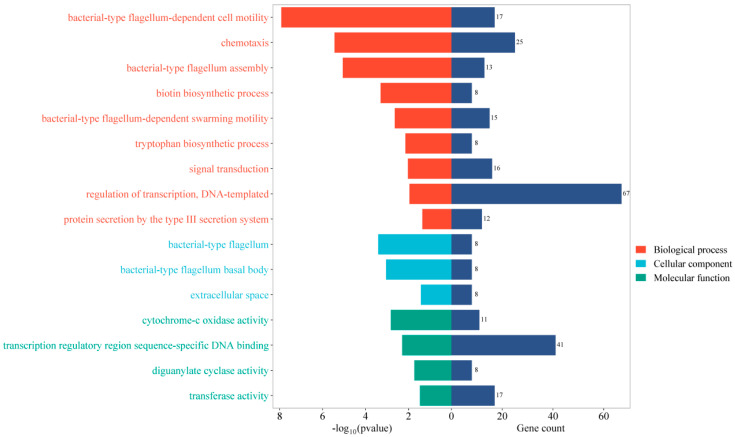
Barplot of GO enrichment analysis. The left side of the figure shows the *p*-value of the GO term (−log10 transformations); the smaller the *p*-value, the longer the bar, and the three parts are differentiated by color. The numbers on the right side of the figure represent the number of genes enriched.

**Figure 3:**
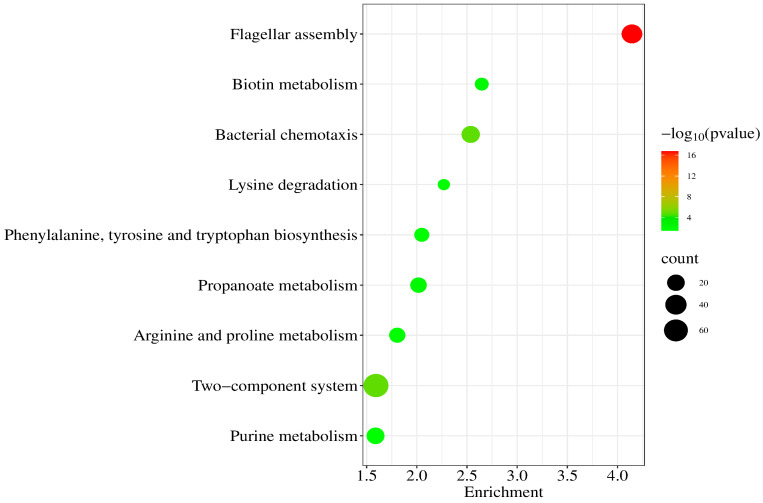
The KEGG pathway enrichment analysis bubble diagram. Bubble size shows the number of genes for each term, while the color reflects the *p*-value, with redder bubbles indicating higher enrichment.

To anticipate the biological function of BF-DEGs, we conducted a functional enrichment analysis. A total of 26 GO terms with *p* < 0.05 were obtained through GO enrichment analysis. In Figure 2, we present only those GO terms with count > 8. Through KEGG pathway enrichment analysis, we identified nine terms with *p*-values < 0.05 and counts > 8. The enrichment results are shown in bubble plots in Figure 3. The highly enriched terms were two-component system, flagellar assembly, and bacterial chemotaxis.

### 3.3. PPI and Identification of the Hub BF-DEGs

**Figure 4:**
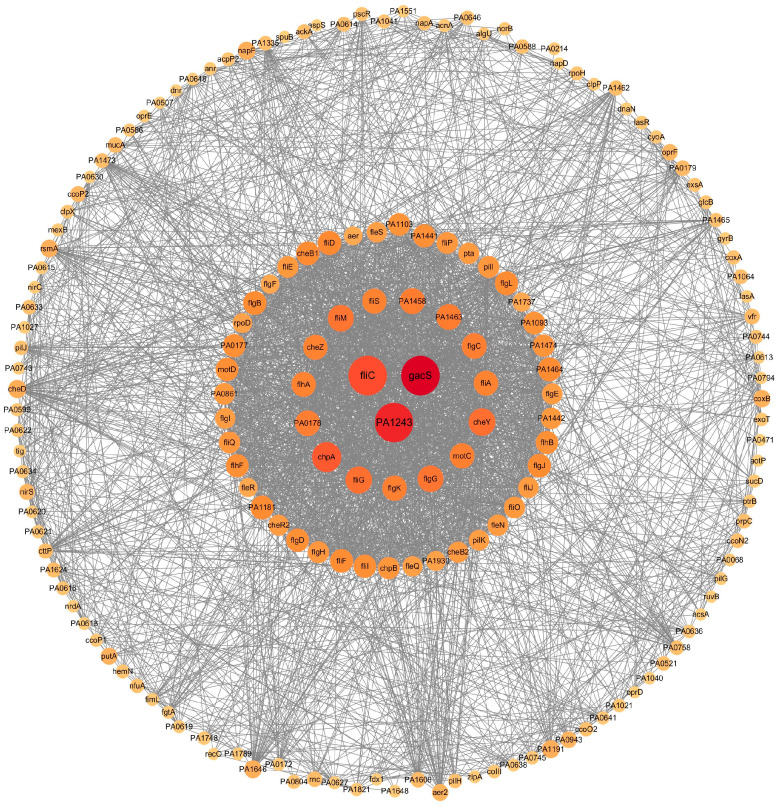
PPI network of the DEGs. Each dot represents a protein, and lines between the dots indicate their interactions. Larger dots correspond to higher node degree values.

**Figure 5:**
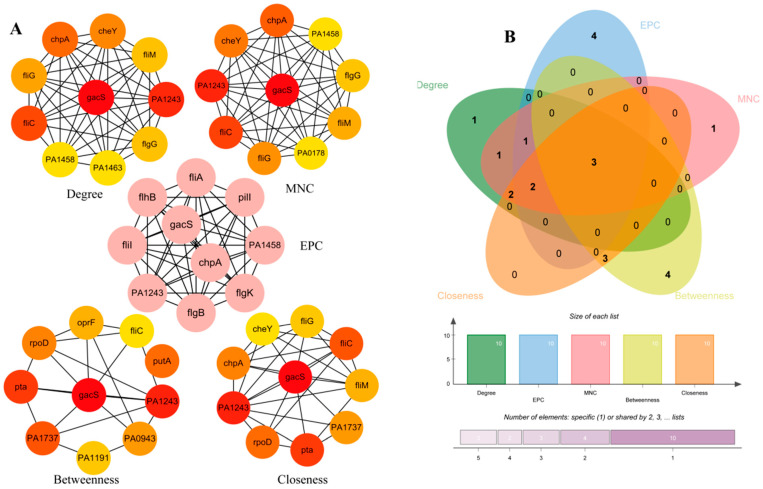
Identification of *gacS* as a key gene for biofilm formation. (**A**) Hub DEGs identified by five algorithms of the Cytoscape plugin cytoHubba. (**B**) Venn diagram showing three overlapping genes in the top 10 genes identified by each of the five topological algorithms.

The PPI network analysis was carried out using the STRING database, which resulted in 1093 nodes and 7108 edges. Each protein represents a target, with larger nodes indicating greater connectivity. Highly connected central nodes may represent key proteins with important physiological roles and could potentially serve as drug targets. Nodes with Degree > 20 were visualized using Cytoscape, as shown in Figure 4. Subsequently, we used five algorithms from the Cytoscape plugin cytoHubba to identify the top 10 genes (Table 2). The intersection of the top 10 hub genes from the five algorithms yielded three overlapping genes (Figure 5): *gacS*, PA1243, and *fliC*. The details of the hub DEGs are presented in Table 2. The key gene *gacS* is the top scorer among these five algorithms, with scores of 124 (Degree), 122 (MNC), 275 (EPC), 82,225 (Betweenness), and 492 (Closeness). Therefore, *gacS* was identified as a central hub gene for biofilm formation; in other words, this gene shows a strong correlation with biofilm formation.

### 3.4. Major Functions and Signaling Pathways of Hub Genes

**Figure 6:**
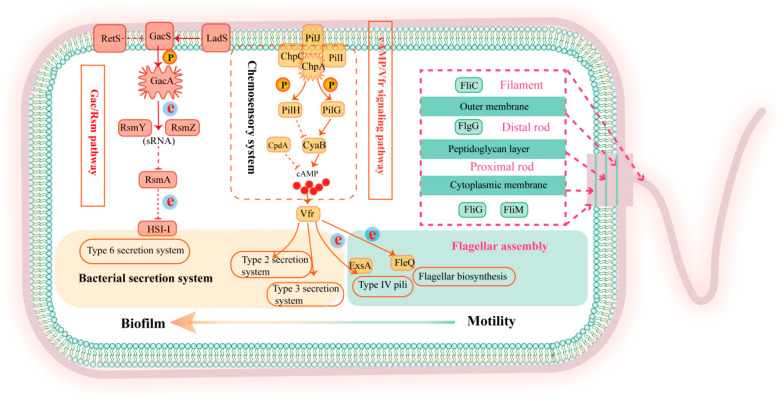
Major functions and signaling pathways of key genes. The activation and inhibition pathways are represented by solid lines and dashed lines, respectively.

The transmembrane sensor kinase GacS and the homologous response regulator GacA form a two-component regulatory system known as the GacS/GacA system [33,34]. GacS can sense extracellular signals and transfer a phosphate group to GacA through autophosphorylation. Once phosphorylated, GacA activates the expression of two small RNAs: RsmZ and RsmY. These small RNAs bind to the transcriptional regulator RsmA, preventing RsmA from repressing its target genes. In this way, the GacS/GacA system regulates the expression of genes associated with biofilm formation, thereby influencing the development of biofilms, as shown in Figure 6 [35,36,37].

The GacS/GacA system plays a crucial role in biofilm formation and antibiotic resistance [38]. The *gacS* mutation alters RsmY/RsmZ transcription, leading to significant suppression of alginate biosynthesis, reduction in biofilm thickness, and increased antibiotic susceptibility [30]. Brinkman et al. found that *gacS* deletion reduces the minimum inhibitory concentrations (MICs) of gentamicin, amikacin, and chloramphenicol in *P. aeruginosa* [39]. The GacS/GacA system plays a pivotal role in activating chronic infection-associated phenotypes by upregulating T6SS expression while suppressing T3SS, thereby driving the bacterial transition toward chronic infection mode [40]. The GacS/GacA two-component system represents a promising target for biofilm regulation. As the histidine kinase (HK) component, GacS serves as the molecular ‘on-switch’ of this signaling cascade, where its kinase activity directly determines GacA phosphorylation status. Targeted inhibition of GacS can therefore effectively disrupt the Rsm regulatory network, which motivated our selection of GacS as the key intervention target in this study.

The cAMP/Vfr pathway regulates virulence factor production in *P. aeruginosa*. cAMP modulates multiple virulence systems, including type IV pili, T3SS, and quorum sensing, through its dependent transcription factor Vfr. The Chp chemosensory system coordinates virulence gene expression and function by regulating CyaB activity to control cAMP levels [41]. Additionally, biofilm formation relies on bacterial motility, such as swimming and twitching, to reach the surface of the medium. Swimming is driven by the rotation of flagella, and twitching motility is powered by type IV pili [42,43,44]. Both flagella and type IV pili provide mechanical and physical support during biofilm formation and colonization, facilitating the ability of the bacteria to transition between acute and chronic infections, thereby adapting to different environmental pressures [45].

In conclusion, the GacS/GacA system is crucial to the physiological activities and infection processes of *P. aeruginosa* by regulating biofilm formation and antibiotic resistance, making it a promising target for therapeutic intervention.

### 3.5. Structure-Based Virtual Screening of FDA-Approved Drugs Identifies Potential GacS Inhibitors

**Figure 7:**
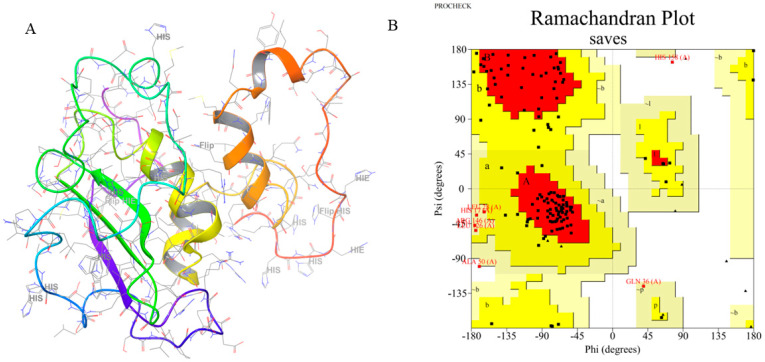
(**A**) Three-dimensional structure of GacS. (**B**) Ramachandran plot analysis of the 13th GacS structural model. The plot shows that the vast majority of amino acid residues fall within the most favored and additionally allowed regions. Generally, a model is considered to have a conformation that satisfies stereochemical rules if more than 90% of its residues are in these regions, which indicates good geometric quality and reliability of the protein structure.

The quality of the ensemble model of the GacS protein structure resolved by the NMR method was assessed via Ramachandran plots. In the conformational ensemble, 94.7% of the residues in the 13th structural model are located in the most favored and additionally allowed regions (as shown in Figure 7), and it has the highest percentage of the most favored regions. The result suggests that the 13th GacS protein structural model is of great quality and suitable for subsequent molecular docking and dynamics simulations.

Docking analysis was conducted on 1729 FDA-approved drugs, targeting the reported binding pocket sites to identify potential GacS inhibitors. A higher negative docking score reflects stronger receptor-ligand affinity, indicating that the compound may exhibit higher potential activity. Following two rounds of SP and XP docking screening, we selected the top 10 compounds with the highest XP Score for cluster analysis to identify the most representative drug candidates. These compounds were subsequently classified into five distinct clusters (Table 3). To further validate the reliability of the virtual screening results and identify compounds with stable binding, the top-ranking candidate drugs from docking were subjected to molecular dynamics simulations.

### 3.6. Molecular Dynamics Simulations

**Figure 8:**
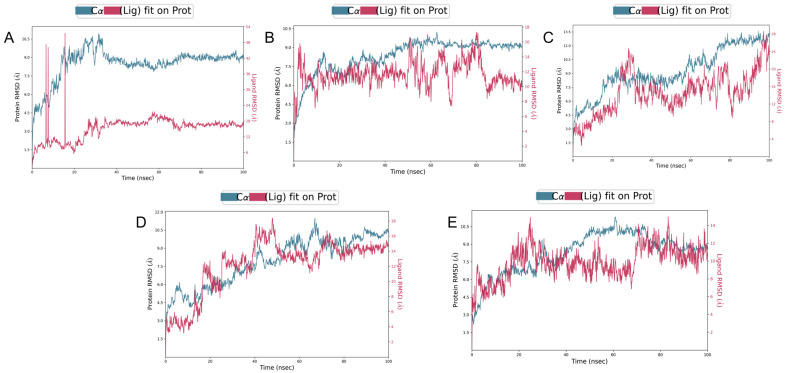
RMSD of protein backbone and ligand. The blue line represents the protein backbone fit to backbone, and the red line represents the ligand fit to backbone. (**A**) Glutathione oxidized (GSSG); (**B**) Arformoterol tartrate (ARF); (**C**) Framycetin sulfate (FRS); (**D**) Desmopressin acetate (DDAVP); (**E**) Lanreotide acetate (LAN).

To explore the stability of binding between the protein and the candidate drugs, we conducted a comprehensive analysis of these interactions using molecular dynamics simulations, as shown in Figure 8.

Firstly, the binding of FRS to the target protein exhibited significant instability throughout the simulation, as reflected by the large fluctuations in the RMSD of both the protein and the ligand. Additionally, the docking score of FRS was relatively low (−6.201), further suggesting that the binding mode may lack strong biological stability.

In contrast, the binding of the other four candidate compounds to the GacS protein demonstrated greater stability. In the case of GSSG, the RMSD of the protein remained relatively stable, indicating minimal global conformational changes. Although the RMSD of the ligand exhibited several significant peaks during the first 20 ns, suggesting noticeable conformational changes or shifts, it gradually stabilized after 40 ns, fluctuating between 15 and 18 Å, and eventually reached equilibrium at approximately 70 ns.

The molecular dynamics simulation of ARF revealed that the protein experienced conformational changes during the first 20 ns, after which it stabilized, with reduced fluctuations, indicating good overall binding stability. The ARF ligand exhibited some volatility in the early stages of the simulation, particularly between 50 ns and 80 ns. This suggests instability in its binding position. After 80 ns, the fluctuations in the RMSD of the ligand remained within 3 Å, indicating that the ligand binding had stabilized.

With respect to DDAVP, the GacS-DDAVP complex showed an increasing trend in both protein and ligand RMSD during the first 80 ns, with significant fluctuations. However, after 80 ns, the fluctuations gradually decreased, indicating that the complex achieved a certain degree of stability in the later stages of the simulation.

Lastly, the RMSD of the LAN ligand showed significant fluctuations during the first 60 ns, indicating that the binding position was not yet stable. However, after 70 ns, the RMSD stabilized between 10 and 13 Å, suggesting that the binding of the ligand to the protein became more stable in the later stages of the simulation.

In summary, the binding interactions of GSSG and ARF with the GacS protein exhibited relatively stable characteristics, with ligand RMSD fluctuations constrained within 3 Å. In contrast, the other ligands displayed greater instability during their binding to GacS. Although these complexes gradually approached equilibrium after approximately 80 ns, their stability remained inferior compared to that of GSSG and ARF, suggesting comparatively less favorable binding modes.

To further analyze these observed differences in binding stability, we subsequently calculated and compared the average binding free energies of these candidate compounds. A lower binding free energy indicates a more stable interaction between the ligand and the protein. Based on the binding free energy results, as shown in Table 4, GSSG and ARF exhibited the lowest binding free energies, at −56.18 and −53.95 kcal/mol, respectively, indicating that they form the most stable interactions with GacS. Furthermore, the relatively small standard deviation of the binding free energy of GSSG suggests that it maintained a consistent binding behavior throughout the simulation. Therefore, these two compounds were selected as candidates for further ligand–protein interaction analysis.

**Figure 9:**
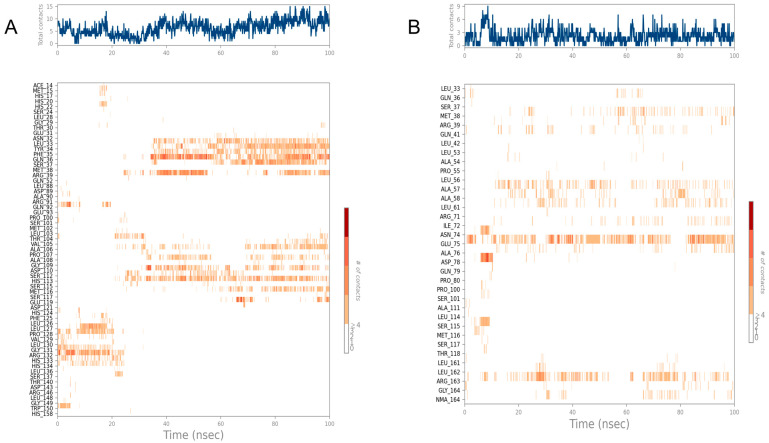
Statistical plot of ligand–protein interaction. (**A**) Glutathione oxidized (GSSG); (**B**) Arformoterol tartrate (ARF).

The binding of GacS with GSSG is more stable compared to its binding with ARF, particularly as equilibrium is reached in a shorter time (approximately 40 ns) with GSSG, whereas ARF requires a longer time to stabilize, with slightly greater fluctuations during the binding process. After 40 ns, GSSG formed very stable interactions with the LEU33 and GLN36 residues, including hydrogen bonds and water-bridged interactions (Figure 9). Additionally, HIS113 interacts with GSSG through hydrogen bonds, water-bridged interactions, ionic bonds, and hydrophobic interactions. These residues play crucial roles in stabilizing the GSSG conformation. Furthermore, after 60 ns, TYR34 and SER37 gradually form hydrogen bonds and water-bridged interactions with GSSG, and SER37 also forms ionic bonds. These interactions, particularly the hydrogen bonds and water-bridged interactions, are key factors in the transition of the ligand from its initial to its stable conformation, as they gradually form in the later stages of the simulation.

In contrast, ARF exhibited greater fluctuations during its binding to GacS but eventually stabilized in the later stages of the simulation. The key residues maintaining the stable binding of ARF are GLU75 and ARG163. GLU75 forms hydrogen bonds, water-bridged interactions, and ionic interactions with ARF, and ARG163 maintains stable contact with the ligand through hydrogen bonds, water-bridged interactions, and hydrophobic interactions. These interactions persisted throughout the simulation and played a critical role in forming the stable conformation of ARF.

Although molecular docking and dynamic simulations identified four candidate compounds (GSSG, ARF, DDAVP, and LAN), we selected GSSG and ARF as the most promising inhibitors for further in vitro validation. This decision was based on their relatively stable binding behaviors demonstrated by smoother RMSD trajectories, minimal protein backbone fluctuations, and lower binding free energies with smaller standard deviations compared to DDAVP and LAN. Therefore, GSSG and ARF were prioritized for subsequent biological experiments.

### 3.7. Minimum Inhibitory Concentration (MIC) of Candidate Drugs and Antibiotics

To rule out the possibility that the compounds reduce biofilm formation indirectly by inhibiting bacterial growth, we first assessed the antibacterial activity of the candidates, as shown in Table 5. Subsequently, we evaluated their anti-biofilm activity at sub-minimum inhibitory concentrations (sub-MIC) using the crystal violet (CV) assay.

### 3.8. Anti-Biofilm Efficacy of Drug Candidates Alone and in Combination with Antibiotics

**Figure 10:**
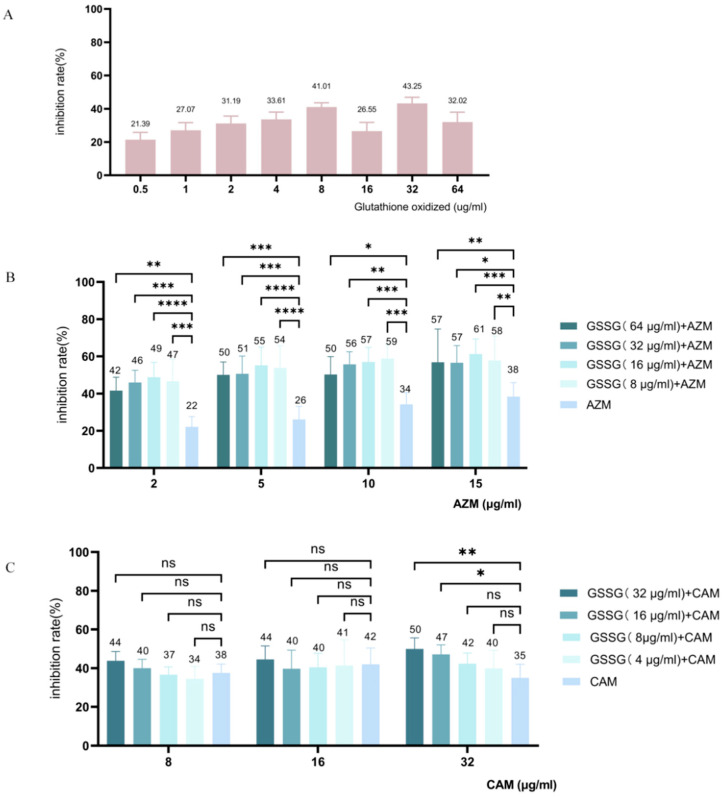
Biofilm inhibition rate of GSSG alone (**A**), combination of GSSG and AZM (**B**), and combination of GSSG and CAM (**C**). Vertical error bars represent the standard error for each group, and the numbers above each bar indicate the mean inhibition rate for that group. The different colors represent varying concentrations of GSSG combined with AZM or CAM. AZM, azithromycin; CAM, clarithromycin; * *p* ≤ 0.05, ** *p* ≤ 0.01, *** *p* ≤ 0.001, **** *p* ≤ 0.0001, ns > 0.5.

The crystal violet staining assay was applied to evaluate the effect of the candidates (alone and in combination with the two antibiotics at sub-MIC doses) on biofilm formation in *P. aeruginosa*. The results showed that GSSG and ARF exhibited anti-biofilm activity. As shown in Figure 10, the results indicate that the biofilm inhibition rate increases with GSSG concentrations ranging from 0.5 μg/mL to 8 μg/mL. When the GSSG concentration was 8 μg/mL, the biofilm inhibition rate reached 41.01%.

After evaluating the inhibition effect of GSSG alone, we further investigated its anti-biofilm activity in combination with sub-MIC concentrations of AZM and CAM. The sub-MIC concentrations of AZM were: 2, 5, 10, and 15 µg/mL. The biofilm inhibition rates were 42–49% for AZM (2 μg/mL) + GSSG, 50–55% for AZM (5 μg/mL) + GSSG, 50–59% for AZM (10 μg/mL) + GSSG, and 57–61% for AZM (15 μg/mL) + GSSG. In particular, the treatment containing 2 µg/mL AZM resulted in a biofilm inhibition rate of 22%. The combination with 16 µg/mL of GSSG resulted in a biofilm inhibition rate of 49% (*p* < 0.0001), which is 2.21 times higher than that of AZM alone, indicating that the combination therapy has a significantly stronger effect than using AZM alone. Notably, the combination of 15 μg/mL AZM and 16 μg/mL GSSG showed the highest biofilm inhibition rate of 61%.

When combining sub-MIC concentrations of GSSG with CAM (at 8, 16, and 32 µg/mL), higher concentrations of GSSG yielded enhanced anti-biofilm activity. Specifically, the combination of CAM with 32 µg/mL of GSSG resulted in the greatest inhibition effect (50%, *p* < 0.01), reflecting a 1.43-fold increase in inhibition rate compared to that obtained at 32 µg/mL CAM alone. However, when GSSG was combined with low concentrations of CAM (8 μg/mL and 16 μg/mL), the enhancement in biofilm inhibition was not significant, with most combinations failing to reach statistical significance (*p* > 0.05). Overall, the combination of GSSG and AZM exhibited a stronger enhancement in biofilm inhibition compared to the results from the combination of GSSG and CAM.

**Figure 11:**
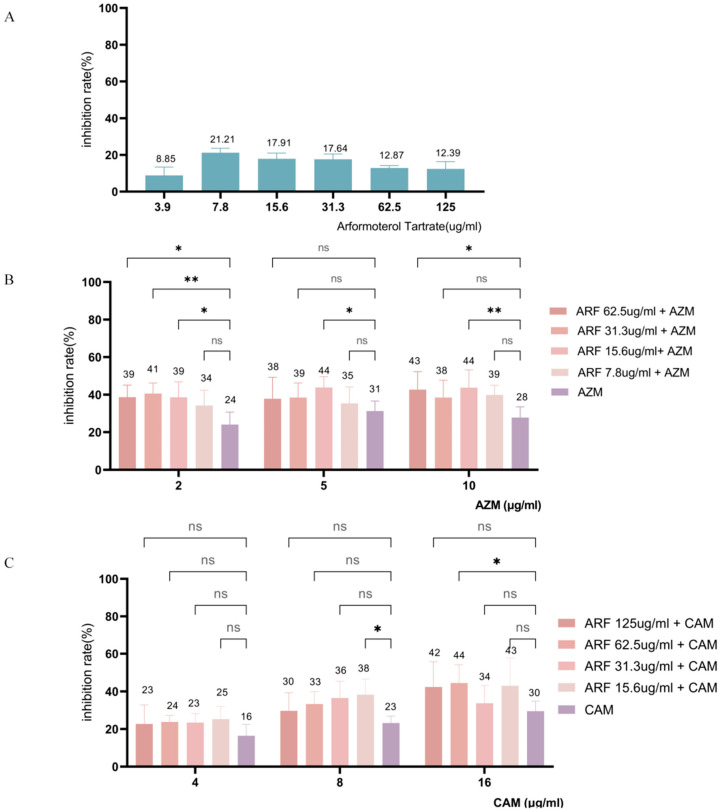
Biofilm inhibition rate of ARF alone (**A**), combination of ARF and AZM (**B**), and combination of ARF and CAM (**C**). The vertical error bars represent the standard error for each group, and the numbers above each bar indicate the mean inhibition rate for that group. The different colors represent varying concentrations of ARF combined with AZM or CAM. AZM, azithromycin; CAM, clarithromycin; * *p* ≤ 0.05, ** *p* ≤ 0.01, ns > 0.5.

As shown in Figure 11, ARF exhibited some anti-biofilm activity when diluted two-fold in the concentration range of 3.9–125 µg/mL. When the concentration of ARF was 7.8–31.3 µg/mL, the biofilm inhibition rate remained stable at 17.64% to 21.21%, indicating a relatively weak inhibitory effect. The maximum inhibition rate of ARF alone was only 21%, which indicates limited efficacy in inhibiting biofilm formation. However, when ARF was combined with AZM or CAM at sub-MIC concentrations, its anti-biofilm activity was significantly enhanced.

When the AZM concentration was 2, 5, or 10 μg/mL, the combination with 15.6 μg/mL ARF showed the greatest inhibitory effect, significantly increasing the inhibition rate of AZM. It is particularly worth noting that when 2 μg/mL of AZM was combined with 31.3 μg/mL of ARF, the inhibition rate of the biofilm increased from 24% to 41% (*p* ≤ 0.01), which was 1.69 times that of AZM alone. This indicates that the combination therapy significantly enhanced the anti-biofilm effect. Among the various combinations of CAM and ARF, the combination of 16 μg/mL CAM and 62.5 μg/mL ARF yielded the highest biofilm inhibition, 44% (*p* ≤ 0.05). However, at lower concentration combinations (4 μg/mL and 8 μg/mL CAM), the addition of ARF failed to significantly enhance the biofilm inhibitory effect of CAM (*p* > 0.05).

In summary, GSSG alone effectively inhibited biofilm formation, while its combination with antibiotics enhanced their anti-biofilm efficacy. Although ARF showed weaker activity when used alone, it significantly potentiated the anti-biofilm effects of low-concentration AZM. These findings provide new perspectives for both the development of anti-biofilm agents and the optimization of combination therapy strategies.

## 4. Discussion

The increased antibiotic resistance and persistence of *P. aeruginosa* are largely linked to biofilm formation [8,46]. Biofilms reduce antibiotic efficacy by limiting drug penetration and altering pH levels, thus enhancing bacterial resistance. Biofilm formation relies on an extensive and intricate regulatory system, controlled by various genes and signaling pathways [16,47]. This complexity is closely linked to the large genome and core-essential genes of *P. aeruginosa*, which provide the bacteria with a flexible adaptability to effectively regulate biofilm formation and maintenance in various environments [48,49]. This study aimed to identify biofilm treatment targets and potential inhibitors through bioinformatics analysis and molecular docking. In vitro experiments evaluated the effectiveness of these inhibitors in combination with conventional antibiotics, offering new insights for the treatment of biofilm-related infections. This research provides strong support for optimizing clinical treatment strategies, particularly in the context of the growing challenge of antibiotic resistance, and holds significant value.

We collected 1098 DEGs from the GEO database. KEGG analysis of the differentially expressed genes indicated that the two-component systems (TCSs) are one of the major enriched pathways. The TCSs enriched in this pathway, such as GacS/GacA, FleS/FleR, ChpA-PilG, and PhoQ/PhoP, regulate the synthesis of factors involved in biofilm formation in *P. aeruginosa*, responding to environmental stimuli and facilitating the transition of bacteria from a motile lifestyle to a sessile one [49,50,51,52]. Our results suggest that TCS-related proteins may play a critical role in the biofilm formation process [53]. Previous studies have shown that the genome of *P. aeruginosa* encodes one of the highest numbers of potential TCSs among bacteria, including 64 sensor kinases, 72 response regulators, and three Hpt proteins [10,48]. This complex sensing and response system enables *P. aeruginosa* to rapidly adapt to environmental changes. Therefore, inhibiting bacterial TCS activity has been suggested as a promising alternative approach for antibiotic therapy [54].

Among the five topological algorithms in cytoHubba, *gacS* scored the highest, indicating a strong correlation with biofilm formation. This finding is consistent with previous experimental studies. Parkins et al. found that biofilm formation was significantly reduced in the Δ*gacA* mutant strain of *P. aeruginosa*, and GacS contains a GacSPD domain composed of 126 residues [55]. Ahmad et al. demonstrated that the *gacS*ΔPD mutant strain exhibited a biofilm formation behavior resembling that of the Δ*gacS*, with a significant reduction in biofilm thickness compared to the PAK strain, accompanied by changes in RsmY/RsmZ transcription [30]. Protein–protein interaction network analysis revealed that *gacS* is closely associated with genes involved in the flagellar assembly pathway, such as *fliC*, *fliG*, *flgG*, and *fliA*. This suggests that the GacS/GacA system may reduce bacterial motility by inhibiting the expression of these motility-related genes, particularly by downregulating the expression of flagellar-associated genes.

Through SP and XP docking in molecular docking, clustering analysis, and evaluation metrics such as RMSD and average binding free energy in molecular dynamics simulations, GSSG and ARF were ultimately identified as potential GacS inhibitors. In the molecular dynamics simulation of GSSG with the protein, we found that the close proximity between the ligand and key residues led to bad contacts, significantly affecting the stability of the simulation. During frames 58–77, some bad contacts were observed between the ligand and residues VAL129, PRO128, and LEU130, with VAL129 being the most severe, followed by PRO128 and LEU130 with fewer contacts. These bad contacts cause increased RMSD fluctuations, compromising the accuracy of the simulation results. Similarly, during frames 148–157, bad contacts were again observed between VAL129, PRO128, and LEU127, further exacerbating RMSD fluctuations. This indicates that the proximity between these residues and the ligand negatively impacted the reliability of the simulation.

Analysis of the molecular dynamics trajectory of ARF revealed that between frames 0 and 105, ARF gradually transitioned from binding the loop region of the protein to the binding pocket, where it formed a stable conformation that persisted until around frame 500. However, during frames 510 to 820, the ligand RMSD exhibited fluctuations. Specifically, between frames 677 and 818, the phenol tail of the ligand left the binding pocket while the head remained inside, resulting in unstable binding. Lastly, in the last 200 frames of the simulation, the binding of the ligand to the protein stabilized (RMSD remaining within 2 Å), indicating that the system had reached a stable conformation. Additionally, between frames 310 and 321 and frames 790 and 812, the carbonyl group of the ligand head formed hydrogen bonds with ALA58, which, although short-lived, contributed to the structural stability. These results suggest that this interaction should be considered in future compound screening.

In summary, analysis of the molecular dynamics trajectories of the two compounds highlights the importance of monitoring the distance between small molecule ligands and key protein residues (VAL129, PRO128, LEU127, LEU130), as well as the formation of hydrogen bonds between the ligand and ALA58. These interactions affect the stability of docking results, the stability of molecular dynamics simulations, and the magnitude of average binding free energy. Specifically, the presence of bad contacts and the formation or absence of hydrogen bonds can influence binding stability and RMSD fluctuations. Therefore, these factors should be given special attention in future screening efforts.

Oxidative stress represents a critical factor in microbial biofilm formation and maintenance. Antioxidants play a pivotal role in biofilm inhibition by scavenging free radicals and mitigating oxidative stress. Glutathione (GSH), one of the most essential antioxidants, participates in cellular antioxidant defense, detoxification, and immune responses. The interconversion between GSH and its oxidized form (GSSG) maintains intracellular redox homeostasis. GSSG is recycled back to GSH through the action of glutathione reductase, which utilizes NADPH as the reducing cofactor, thereby ensuring sustained cellular GSH availability [56,57]. Yani Z et al. reported that both GSH and GSSG can modulate the susceptibility of *P. aeruginosa* to tetracycline [58]. The combination of GSSG with carbapenem antibiotics demonstrated synergistic bactericidal effects in vitro, reducing the MIC by 4-fold (FICI <0.5) against *blaNDM-1*-carrying resistant *E. coli*. In a murine model infected with *blaNDM-1*, this combination therapy not only reduced bacterial loads in the liver and spleen by three orders of magnitude but also significantly decreased infection-related mortality [59]. Further studies by the Klare group revealed that glutathione disrupts approximately 50% of *Pseudomonas aeruginosa* biofilms by neutralizing ROS through its antioxidant activity. The mechanism involves interference with pyocyanin-eDNA interactions, thereby compromising biofilm integrity and reducing bacterial virulence and pathogenicity. Notably, glutathione induces metabolic reactivation in biofilm-associated persister cells, rendering them more susceptible to antibiotic eradication, a characteristic that provides distinct advantages for combination therapies [60]. In this study, the combination of GSSG with AZM significantly enhanced the anti-biofilm efficacy of the antibiotic, particularly at low AZM concentrations (2 μg/mL), increasing the biofilm inhibition rate from 22% (AZM alone) to 49%. In the monotherapy groups, GSSG exhibited the highest biofilm inhibition rate (43%) at a concentration of 32 µg/mL, while AZM alone showed its maximum inhibition rate (38%) at 15 µg/mL. When combining these optimal concentrations of GSSG (32 µg/mL) and AZM (15 µg/mL), the inhibition rate increased significantly to 57%, which is approximately 1.5 times greater than that achieved by AZM alone. These results further support the therapeutic potential of combination treatment, offering a novel strategy for anti-biofilm therapy. This also suggests that GSSG may act as a dual-target inhibitor, potentially neutralizing reactive oxygen species (ROS) on the one hand and inhibiting the GacS/GacA two-component system on the other.

ARF is a long-acting β2-agonist used for long-term maintenance therapy in COPD patients, improving lung function and alleviating respiratory symptoms like wheezing and shortness of breath [61]. COPD induces physiological changes in lung immune defense, often accompanied by respiratory infections [62]. Recurrent infections may progress to chronic infection through biofilm formation, and bacterial infections are likely one of the main causes of acute exacerbations of COPD (AECOPD), with approximately 50% of AECOPD cases being associated with bacterial infections [63,64,65]. AZM has been shown to provide significant clinical benefits for patients with severe COPD, owing to its anti-inflammatory properties, ability to reduce exacerbations, and inhibition of biofilm formation [65,66,67]. Pomares et al. found that long-term intermittent use of AZM as an adjunct to the standard triple therapy regimen (long-acting anticholinergics, long-acting β-agonists, and inhaled corticosteroids) could reduce AECOPD incidence and hospitalization rates by more than half, while significantly shortening hospital stays [65]. The results of our study demonstrated that although ARF demonstrated some biofilm inhibition activity, its effect was relatively limited when used alone, with a maximum inhibition rate of only 21%. This suggests that ARF, as a monotherapy, may not be sufficient for effectively inhibiting biofilm formation in *P. aeruginosa*. The relatively weak inhibitory effect of ARF alone highlights the need for combination therapies to achieve a more potent anti-biofilm effect, which was clearly observed when ARF was combined with antibiotics such as AZM and CAM. As observed in our study, combining 31.3 μg/mL ARF with 2 μg/mL AZM increased the biofilm inhibition rate by 1.69 times compared to AZM alone. The enhanced efficacy of combined treatment (ARF and AZM) may offer a novel therapeutic strategy for biofilm-associated infections in COPD patients. It enhances anti-biofilm efficacy, reduces antibiotic dosage and resistance risks, and simultaneously improves lung function and overall patient outcomes. Therefore, the combination of GSSG and ARF with conventional antibiotics may provide a new clinical treatment strategy for biofilm-related infections. Even a slight enhancement of anti-biofilm effects could have a positive impact on clinical treatment success and patient recovery, especially in the context of increasing antibiotic resistance and the growing challenges in treating biofilm-related infections. In future experiments, we plan to compare the biofilm inhibition rates of monotherapy antibiotics, monotherapy candidates, and combination therapy groups, and calculate fractional inhibitory concentrations (FICs) to determine the indicated synergy, indifference, or antagonism.

The limitation of this study is that, although multiple DEGs were identified through differential gene expression analysis, other genes, such as chpA and cheY, in addition to gacS, also scored highly in five topological algorithms. These algorithms include Degree, EPC, MNC, Betweenness, and Closeness, suggesting that chpA and cheY may also serve as potential targets for biofilm treatment. Therefore, there is still substantial room for further exploration. Another notable limitation is the lack of mechanistic validation regarding the downstream signaling cascade of GacS. We will select two FDA-approved drugs as lead compounds for structural modification, with the aim of developing more potent GacS inhibitors. Additionally, we plan to combine these inhibitors with other antibacterial agents to explore broader combination therapy strategies. It is worth noting that GacS is present not only in *P. aeruginosa* but also in *P. fluorescens* and *P. syringae* [68,69]. This suggests that the combination therapy strategies explored in this study may not only be effective against the target pathogen but also have potential efficacy against other bacterial infections. Further research is needed to assess its effectiveness in broader clinical applications and offer new solutions for treating bacterial infections.

## 5. Conclusions

In this study, we identified GacS as a promising therapeutic target for biofilm formation in *P. aeruginosa* through bioinformatics analysis. Through virtual screening and molecular dynamics simulations, we identified that glutathione oxidized (GSSG) and arformoterol tartrate (ARF) are potential GacS inhibitors. In vitro experiments confirmed that these two compounds exhibited promising anti-biofilm activity, particularly showing enhanced inhibitory effects when combined with azithromycin (AZM) and clarithromycin (CAM). These results suggest that GSSG and ARF may be promising candidate drugs for potentially combating *P. aeruginosa*-related biofilm infections.

## Figures and Tables

**Table 1:** Up- and down-regulated DEGs identified through integrated data.

DEGs	Gene Symbol
Up-regulated	PA0165 PA0975 *metX* PA0087 *pscP* PA0169 PA1855 PA1020 PA1271 *kefB* PA0040 PA0951 PA0046 PA0578 *pscR* PA1689 *estX* PA1237 PA0086 PA1274 *tig micA* PA0244 PA1845 *sdhD cobB* PA1302 PA1922 PA0172 *cobC* PA0170 *thiG* PA1791 PA0380 *aspS* PA0277 PA0202 PA0045 PA1918 *cobQ*
Down-regulated	PA1414 PA1673 PA1746 PA0960 PA0713 PA0109 *gcdH* PA0656 *aer* PA0141 PA1429 PA0745 PA0744 PA1076 *cheY* PA1745 *aer2* PA0449 PA1474 PA1749 PA1736 PA1245 PA1041 PA0565 *rpoH* PA0586 PA0250 PA0177 *mvfR* PA1860 PA0484 PA1117 PA1789 *xdhA* PA0588 *ackA napD fleQ* PA1517 *napF*

**Table 2:** The top 10 hub genes ranked by five algorithms in cytoHubba.

Degree	EPC	MNC	Betweenness	Closeness	Overlap
*gacS*	*gacS*	*gacS*	*gacS*	*gacS*	*gacS*
PA1243	*fliS*	PA1243	PA1243	PA1243	PA1243
*fliC*	*fliC*	*fliC*	*pta*	*pta*	*fliC*
*chpA*	PA1458	*chpA*	PA1737	*fliC*	
*cheY*	*flgC*	*cheY*	*putA*	*rpoD*	
*fliG*	*cheY*	*fliG*	*rpoD*	*chpA*	
*fliM*	PA1243	*fliM*	PA0943	PA1737	
*flgG*	*fliA*	*flgG*	*oprF*	*fliM*	
PA1458	*flhB*	PA1458	PA1191	*fliG*	
PA1463	*chpA*	PA0178	*fliC*	*cheY*	

**Table 3:** The results of cluster analysis.

Cluster	Drug Candidates	CAS	Docking Score
Cluster 1	Glutathione oxidized (GSSG)	27025-41-8	−7.905
Cluster 2	Arformoterol tartrate (ARF)	200815-49-2	−6.803
Cluster 3	Framycetin sulfate (FRS)	4146-30-9	−6.201
Cluster 4	Desmopressin acetate (DDAVP)	62288-83-9	−7.900
Cluster 5	Lanreotide acetate (LAN)	2378114-72-6	−6.777

**Table 4:** Binding free energy of drug candidates.

Drug Candidates	dG Average	dG Standard Deviation
Glutathione oxidized (GSSG)	−56.1831	4.26
Arformoterol tartrate (ARF)	−53.9521	5.97
Desmopressin acetate (DDAVP)	−41.5773	5.25
Lanreotide acetate (LAN)	−48.6991	4.76

**Table 5:** MIC values of candidates and antibiotics.

Drug	MIC
GSSG	>256 μg/mL
ARF	>256 μg/mL
AZM	32 μg/mL
CAM	64 μg/mL

## Data Availability

Data presented in this study are available on request from the corresponding author. The data are not publicly available due to the samples in this study being secondary specimens from clinical patients.
